# Infant Social Avoidance Predicts Autism but Not Anxiety in Fragile X Syndrome

**DOI:** 10.3389/fpsyt.2019.00199

**Published:** 2019-05-07

**Authors:** Jane E. Roberts, Hayley Crawford, Elizabeth A. Will, Abigail L. Hogan, Samuel McQuillin, Bridgette L. Tonnsen, Shannon O'Connor, Douglas A. Roberts, Alexis M. Brewe

**Affiliations:** ^1^Department of Psychology, University of South Carolina, Columbia, SC, United States; ^2^Centre for Innovative Research Across the Life Course, Coventry University, Coventry, United Kingdom; ^3^Department of Psychological Science, Purdue University, West Lafayette, IN, United States; ^4^Georgia State University, Atlanta, GA, United States

**Keywords:** social avoidance, FMR1, fragile X syndrome, autism, anxiety

## Abstract

**Objective:** Autism spectrum disorder (ASD), attention-deficit/hyperactivity disorder (ADHD), and anxiety are three of the most common childhood psychiatric disorders. Early trajectories of social avoidance have been linked with these psychiatric disorders in previous studies, but it remains unclear how social avoidance differentially predicts comorbid disorders in a high-risk genetic subgroup. Here, we delineate the association between trajectories of social avoidance from infancy and subsequent ASD, ADHD, and anxiety outcomes at preschool in children with fragile X syndrome (FXS), a well-characterized single-gene disorder highly associated with social avoidance as well as elevated rates of ASD, ADHD, and anxiety.

**Method:** Males with FXS (*n* = 78) aged 4–62 months participated in a longitudinal study resulting in 201 assessments. The Social Avoidance Scale (SAS) documented socially avoidant behaviors from infancy in three domains—physical movement, facial expression, and eye contact during both the first minute and the last hour of an interaction. ASD, ADHD, and anxiety symptom outcomes at preschool were measured via parent-report questionnaires.

**Results:** Increased social avoidance across infancy and preschool predicted elevated ASD symptom severity but reduced ADHD and anxiety symptom severity in males with FXS.

**Conclusion:** ASD, ADHD, and anxiety symptoms relate inconsistently to social avoidance behaviors, providing new insight toward the debate of independence or overlap among these disorders in FXS and other disorders (i.e., ASD). The results suggest that the nuanced profile of the developmental and temporal aspects of social avoidance may inform more the accuracy of differential diagnoses of comorbid psychiatric disorders in FXS.

## Introduction

Psychiatric disorders are debilitating and common, affecting more than one in five children (1). Autism spectrum disorder (ASD) is one of the most common and impairing disorders affecting nearly 2% of the general population (2). The diagnosis of ASD is complicated, however, given the common co-occurrence of other disorders. Attention-deficit/hyperactivity disorder (ADHD) and anxiety are two psychiatric disorders that often present in children with ASD. Differential diagnosis of ASD, ADHD, and anxiety in young children is challenging given the subtlety of behavioral features in early development coupled with the presence of features that represent typical variation or overlap across multiple disorders. Adopting a developmental trajectory approach to capture variation linked to the emergence of psychiatric disorders will increase understanding of the timing and targets for both prevention and intervention (3). As such, studies that identify precursors or symptoms of ASD, ADHD, and anxiety in infancy and track these symptoms over time until the age of diagnosis hold great promise to advance psychiatric research. Of interest to this study, social avoidance is a core or associated feature of ASD, ADHD, and anxiety that often presents early in development (4, 5). Identifying developmental trajectories of social avoidance in groups at high risk for ASD, ADHD and anxiety is likely to advance our understanding of both the emergence and independence of features across these common and impairing psychiatric disorders.

While mean levels of symptom impairment may index risk for psychiatric disorders, clear evidence suggests that developmental trajectories representing change or stability can be uniquely predictive (4, 6). For example, infants later diagnosed with ASD display reduced avoidance at 6 months-of-age followed by increased levels of avoidance that emerge at 12 months-of age and continue through preschool (7, 8). Similarly, trajectories of change in avoidance and reactivity across the first years, but *not* mean levels at any specific age, predict ASD outcomes (9). Also, a stable trajectory of reduced avoidance from infancy through preschool characterize children later diagnosed with ADHD (10, 11). Finally, both high and stable levels, as well as steep increases of social avoidance and stranger fear from 6 to 36 months-of-age, has been linked to elevated risk for anxiety in preschool (6, 12) through to middle childhood (4).

Whereas most studies of social avoidance have focused on “typically developing” children, examining social avoidance trajectories in genetic subgroups at elevated risk for ASD, ADHD, and anxiety may inform biological influences on symptom emergence, as well as the generalizability of social avoidance research conducted in non-clinical samples to clinical groups. Fragile X syndrome (FXS) is a well-characterized single-gene disorder highly associated with co-occurring features of ASD, ADHD, and anxiety, making the disorder an ideal candidate for studying social avoidance trajectories in a “high risk,” genetically-defined sample. FXS is the most common inherited cause of intellectual disability (ID), affecting ~1 in 4,000 males and 1 in 8,000 females (13). FXS is caused by a cytosine-guanine-guanine (CGG) expansion and subsequent methylation of *FMR1* gene on the Xq27.3 site resulting in reduced FMRP, the protein associated with FXS. Males with FXS are typically more severely affected given random X inactivation in females associated with elevated FMRP. Along with moderate ID, 50–70% of males with FXS meet criteria for ASD (14, 15) and 53–73% meet criteria for ADHD (16, 17). Anxiety is also highly prevalent, affecting over 85% of males (18). In addition, social avoidance—a feature also associated with ASD, ADHD, and anxiety—is a hallmark characteristic of 82–98% of males with FXS (19–21). The phenotypic overlap of FXS with ASD, ADHD, and anxiety offers an ideal model for understanding gene-brain-behavior relationships in a simplified genetic context (22).

The validity and independence of ASD, ADHD, and anxiety disorders in FXS has been challenged, not surprisingly, with debate regarding whether they represent “true” comorbidities or whether co-occurring symptomology shared across these disorders results in artifactual diagnostic categorization (21, 23). For example, poor eye contact is a nearly universal feature of FXS (24–26); yet, it is not clear whether poor eye contact indexes the presence of ASD or anxiety, both ASD and anxiety or neither ASD nor anxiety in FXS. These questions of “true” diagnoses and the confounding association of anxiety symptomatology and social avoidance are also debated in the non-syndromic ASD field (27, 28). Exploring the trajectory of specific traits that are common across disorders can provide valuable insight into the distinction and potential overlap among multiple psychiatric outcomes.

Social avoidance is a core or associated feature of ASD, ADHD, and anxiety and is a hallmark characteristic of males with FXS (25, 26). In a recent study (29), we confirmed initial reports that social avoidance in males with FXS is elevated throughout infancy and early childhood relative to age-matched typically-developing control subjects, with 73% of males with FXS exhibiting social avoidance. Furthermore, this work suggests that social avoidance in males with FXS emerges in infancy and increases in severity across early childhood, becoming more stable in adolescence and early adulthood. However, little work has focused on the relationship of social avoidance to the symptoms of ASD, ADHD, and anxiety across development. In the current study, we extend previous work (25, 26) to characterize the association between trajectories of social avoidance and subsequent ASD, ADHD, and anxiety outcomes in FXS from infancy through preschool. ASD and ADHD are two of the earliest emerging childhood disorders, typically presenting before 5 years of age (30–32). In contrast, anxiety disorders are most commonly identified during late childhood and adolescence (33). However, features of anxiety are evident during the infant and preschool years, and anxiety can be diagnosed in preschool-aged children (34). Given clear evidence that ASD, ADHD, and anxiety have optimal outcomes when recognized and treated early (35–37), our focus on the very early years of life is critical for maximal translational effects.

Using a prospective longitudinal design, we examined the relationship between trajectories of social avoidance from infancy to the severity of ASD, ADHD, and anxiety outcomes at preschool in males with FXS. We hypothesize that males with FXS who exhibit higher ASD and anxiety symptoms will exhibit higher levels of social avoidance that will intensify across age. This relationship will not be present in ADHD outcomes. This hypothesis is based on the fact that social avoidance is a core feature of both ASD and anxiety, whereas in ADHD, social avoidance may present as a secondary product of attention-related difficulties in managing social situations. Distinguishing the relationship of the level and trajectories of social avoidance to ASD, ADHD, and anxiety in FXS will provide novel information about their differential impact on these three psychiatric disorders that occur frequently and are very impairing in this population. As such, this work is critical to improve differential diagnostic processes and direct targeted treatment.

## Methods

### Participants

Participants were drawn from two sites that conducted longitudinal studies and included the measures reported here (University of South Carolina and University of North Carolina, Chapel Hill). Participant's parents at all sites provided written informed consent prior to participation. All of these studies focused on documenting the phenotype of FXS across early development with identical procedures and a subset of common measures. The age of the samples, however, varied across studies with some focused on infants while others focused on preschoolers. Participants were drawn from these studies if they were male, under 6 years of age and had at least one assessment that included all of the measures listed below. The advantage of including data from multiple studies allows us to generalize across contexts so our findings are not restricted to one discrete age group and to increase the sample size given the relatively low prevalence of FXS. All participants with FXS were recruited from past studies, national parent listservs, social media, the National Fragile X Foundation, and the Research Participant Registry Core of the Carolina Institute for Developmental Disabilities at the University of North Carolina at Chapel Hill. The full mutation of the *FMR1* gene (>200 CGG repeats) was confirmed through genetic testing. Subsets of these data have been published previously (25, 26); however, data including this large sample for the SAS and the inclusion of three psychiatric outcomes have not been published.

From the potential pool of participants, 74 males with FXS representing 201 assessments met criteria for study inclusion. Chronological age at the first assessment ranged from 4 to 62 months (*M* = 27.31 months, *SD* = 15.12) while chronological age at the outcome assessment ranged from 23.28 to 70.10 months (*M* = 51.81, *SD* = 13.22). The average time between the first and final assessment was 29.39 months (range of 11.04 to 59.64 and SD = 12.10). Nine participants only had one assessment, whereas the rest (*n* = 65) had 2 or more assessments.

### Measures

#### Social Avoidance Scale

The Social Avoidance Scale [SAS; (25, 26)] is an experimental observation scale used to document socially avoidant behaviors in three domains: physical movement, facial expression, and eye contact. Higher ratings represent more socially avoidant behaviors. Two ratings are assigned for each of the three SAS subscales, the first rating is based on the first *minute* of social interaction and the second rating is based on the *last hour* of structured social interaction. Parents were present for both periods of interaction. This results in six SAS ratings (three first-minute and three last hour). Intra-class correlation coefficients (ICCs) reflect a moderate to high degree of reliability between raters on all of the SAS scales, with a range from 0.82 to 0.90. The capacity for the SAS to capture dynamic aspects of social avoidance is central given evidence that a decrease in avoidance between the two ratings is a core feature of FXS and one that differentiates those with and without comorbid ASD (25, 26). The SAS was completed at every assessment point, and the ratings taken at the first minute and last hour for each of the three domains were used as the independent variables to predict ASD, ADHD, and anxiety symptom severity at the final assessment within each age cohort.

#### Autism Spectrum Disorder (ASD) Symptom Severity

ASD severity was measured using the Childhood Autism Rating Scale [CARS; (38)], an observational rating scale comprised of 15 items that measures specific ASD features, such as non-verbal and verbal communication and adaptation to change, for individuals from 2 years of age. While the Autism Diagnostic Observation Scale-2 [ADOS-2; (39)] is recognized as the most robust diagnostic measure of ASD, we utilized the CARS for this study as we were focused on ASD symptoms and not diagnoses and only a subset (~40%) had an ADOS-2 while all participants had a CARS. Also, we have documented a strong relationship between CARS ratings and ADOS-2 continuous severity scores in males with FXS [*r* = 0.90; (40)]. In our sample, 48.72% had CARS raw scores of 30 or above suggesting the presence of ASD. The total raw score of the CARS at the final assessment was used as the dependent variable in the ASD models.

#### Attention Deficit/Hyperactivity Disorder (ADHD) Symptom Severity

The ADHD Problems scale of the CBCL was utilized to measure ADHD symptoms. The ADHD subscale assesses symptoms including inability to sit still, talking too much, and failing to concentrate for long periods of time. In our sample, 56.41% had scores above the clinical cutoff suggesting a high likelihood of ADHD. Due to potential floor and ceiling effects in standardized scores and the fact that the t-scores are restricted to 50 and above, the raw score of the CBCL ADHD Problems at the final assessment was used as the dependent variable in the ADHD models.

#### Anxiety Symptom Severity

Anxiety symptoms were measured using the DSM Anxiety Problems scale of the Child Behavior Checklist [CBCL; (40)], a parent questionnaire assessing emotional and behavioral problems in children. Parents completed the CBCL version appropriate for their child's age (CBCL 1.5–5 Years; CBCL 6–18 Years). The DSM Anxiety Problems scale assesses symptoms associated with anxiety, such as worries, fears about going to school, and clinging to adults. In our sample, 7.70% had scores above the clinical cutoff suggesting a high likelihood of having anxiety. Due to potential floor and ceiling effects in standardized scores and the fact that the *t*-scores are restricted to 50 and above, the raw score of the CBCL Anxiety Problems scale at the final assessment was used as the dependent variable in the anxiety models.

#### Developmental Level

The Mullen Scales of Early Learning (41) is a standardized developmental measure of abilities for children from birth to 68 months. The Early Learning Composite (ELC), an overall estimate of cognitive functioning, was used to analyze the effect of developmental level on measures of social avoidance, ASD, ADHD, and anxiety.

### Procedure

A standard battery of direct child assessments that included developmental and temperament assessments and parent/caregiver rating scales was administered by trained personnel over the course of 2 days [see (42, 43) for more details]. To control for potential effects associated with variation in the assessments across sites and given our primary interest on initial social avoidance, this study focused on SAS ratings for Day 1 only. Across all sites, the SAS and developmental measures were completed at each assessment. All available SAS ratings were included to generate the trajectories of social avoidance, and the ASD, ADHD, and anxiety outcome data were restricted to the single assessment at the child's oldest age. All procedures were implemented with approval from the institutional review boards at each respective site.

### Data Analytic Plan

**Figure 1:**
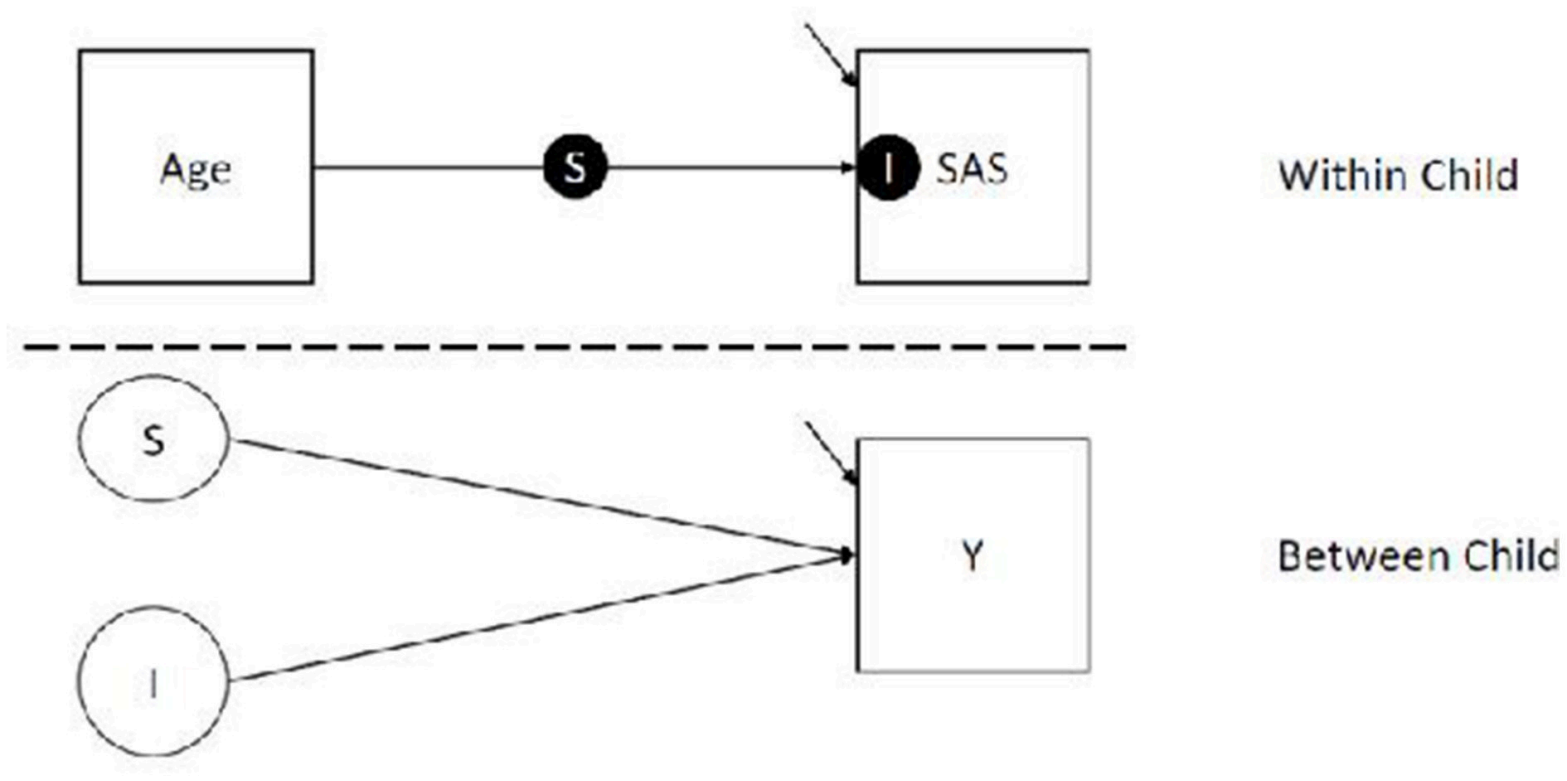
Model of analytic plan.

Analyses were conducted using SPSS 24 (44), R Core Team (45), and Mplus Version 8 (46). As preliminary analyses, we calculated Pearson correlations between ASD, anxiety, and ADHD symptoms, controlling for developmental level, to coarsely assess overlap among symptom profiles and inform structure of subsequent models. Results indicated no relationship between CARS and both anxiety (*r* = −0.004; *p* = 0.972) and ADHD (*r* = 0.170; *p* = 0.162). Anxiety and ADHD were moderately related (*r* = 0.240; *p* = 0.047). Thus, separate models were used for each outcome. We analyzed our primary research questions using random effect latent variable models, which account for variable assessment intervals. Specifically, we used 2-level random slope and intercept model with continuous child-level outcomes. The two levels of our model correspond to (1) variation within child (i.e., child observations across time nested within child) and (2) variation between children. Our model is represented in Figure 1.

In the within portion of our model (i.e., Level 1), we regressed social avoidance measures (i.e., SAS) on the age of the child to estimate a) a latent slope (i.e., **S**, the solid circle on top of the line seen in Figure 1 the within portion of the model) corresponding to the average unit increment in social avoidance per standard deviation unit increase in age, and b) a latent intercept (i.e., **I**, the solid circle within the SAS box in Figure 1) corresponding to the social avoidance at the average age. We grand mean centered and scaled Age to a variance of 1. We used the grand mean to center because children were measured at different times, and some children completed more assessments than others; because of this, interpretation of child centered (i.e., centering on level 2 clusters) age means would be difficult to interpret. In our models, the intercept is interpreted as the predicted social avoidance for a child at the average age of our sample, which is 36.12 months.

In the between portion of our model, we regressed the aforementioned random slope (the unfilled circle S in Figure 1) and intercept (the unfilled circle I in Figure 1) on the child-level outcome (square box Y in Figure 1), which includes measures of ASD, ADHD, or anxiety at the final assessment. In these models, the regression lines correspond to the average difference in our outcome measures per unit difference in the slope and intercept. This portion of the model answers both of our research questions, with question 1 (testing the influence of levels) being answered by regression of child-level outcome on the latent intercept, and question 2 (testing the influence of linear trajectory) being answered by the regressing child-level outcomes on the latent slope of SAS score by Age.

## Results

### Describing Intercepts and Slopes

**Figure 2:**
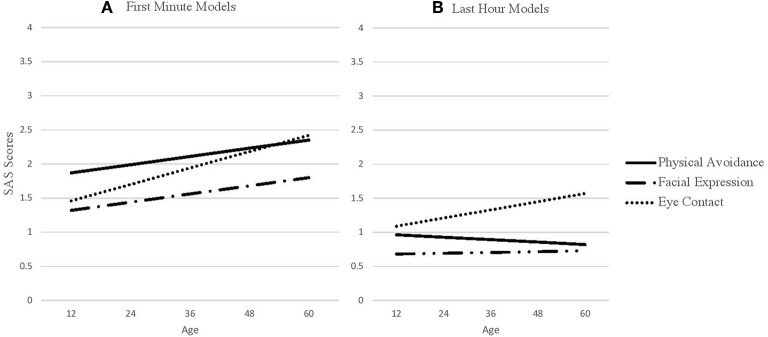
SAS predictors across age. **(A)** SAS first minute predictors across age. **(B)** SAS last hour predictors across age.

Prior to completing the full models, we first estimated global (i.e., independent of outcome) slopes and intercepts for each SAS predictor (physical movement, facial expression, and eye contact) for the first minute and last hour of interaction. These global scores are found in Table 1. These scores are constructed using month-unit measures of age (i.e., as opposed to scaled age as in our full model). The interpretation of the intercept is the average SAS score at 36.12 months, which is the average age of the sample. The slope is the average linear difference in social avoidance for every month increase in Age. Base model results are presented in Table 2 and base model trajectories are presented in Figure 2.

**Table 1:** Demographics table.

	**Mean (SD)**
Age at initial assessment	27.31 (15.12)
Mullen early learning scales composite standard score	58.34 (13.93)
Autism symptoms: childhood autism rating scale-II total raw score	28.64 (6.62)
ADHD^†^ symptoms: child behavior checklist, DSM scale, T score	63.16 (8.67)
Anxiety symptoms: child behavior checklist, DSM scale, T score	54.23 (6.23)
**Race**	**%**
Caucasian	77.00
African American	5.12
Hispanic or Latino	1.30
Asian	0
American Indian/Alaska Native	1.30
Bi-Racial	15.40

† *ADHD, attention deficit hyperactivity disorder*.

**Table 2:** Base model results.

	**Intercept**	**95% CI**		**Slope**	**95% CI**	
		**Lower**	**Upper**		**Lower**	**Upper**
**FIRST MINUTE**						
Physical movement	2.24	2.10	2.40	0.01	0.01	0.02
Facial expression	1.67	1.50	1.84	0.01	0.004	0.02
Eye contact	2.02	1.74	2.30	0.02	0.01	0.04
**LAST HOUR**						
Physical movement	0.90	0.67	1.10	−0.003	−0.013	0.01
Facial expression	0.70	0.55	0.85	0.001	−0.01	0.01
Eye contact	1.33	1.10	1.60	0.01	−0.001	0.02

### Estimating the Influence of Slopes and Intercepts on Outcomes

In our full models, age and SAS scores were scaled to a mean of zero and variance of one. Results from the full models supported our hypotheses that SAS levels and trajectories would predict ASD outcomes. However, our hypothesis that SAS levels and trajectories would predict anxiety and ADHD outcomes was only partially supported (see Table 3 for full results).

**Table 3:** Slopes and intercepts models.

	**Intercept**	***z***	**CI lower**	**CI upper**	**Slope**	***z***	**CI lower**	**CI upper**
**CARS**								
**First minute**								
Physical movement	−2.05	−0.23	−19.44	15.34	39.24	1.14	−28.12	106.61
Facial expression	3.16^**^	10.12^**^	2.55	3.77	51.47^**^	355.02^**^	51.19	51.75
Eye contact	8.15^*^	3.23^*^	3.20	13.10	21.39	1.40	−8.46	51.24
**Last hour**								
Physical movement	5.84^**^	3.51^**^	2.58	9.12	7.93^∧^	2.17^∧^	0.76	15.12
Facial expression	8.51^*^	3.41^*^	3.61	13.40	15.39	1.10	−11.92	42.70
Eye contact	10.05^**^	4.97^**^	6.08	14.02	8.22^**^	3.73^**^	3.90	12.55
**ADHD**								
**First minute**								
Physical movement	−4.68	−1.64	−10.28	0.92	−10.787^∧^	−2.89^∧^	−18.10	−3.47
Facial expression	−0.48	−0.45	−2.55	1.60	−0.80	−0.09	−18.86	17.27
Eye contact	−0.30	−0.38	−1.83	1.24	−0.58	−0.37	−3.70	2.53
**Last hour**								
Physical movement	−0.03	−0.06	−1.08	1.01	1.95	1.55	−1.08	5.00
Facial expression	0.36	0.53	−0.96	1.67	1.45	0.321	−7.42	10.33
Eye contact	0.39	0.54	−1.01	1.79	1.61	0.50	−4.66	7.88
**ANXIETY**								
**First minute**								
Physical movement	0.83^∧^	0.47^∧^	−2.61	4.26	−11.33	−2.07	−22.06	−0.61
Facial expression	0.86	0.73	−1.44	3.17	−0.005	−0.001	−13.66	13.65
Eye contact	0.18	0.29	−1.01	1.36	1.31	0.61	−2.88	5.50
**Last hour**								
Physical movement	−0.03	−0.07	−0.77	0.72	0.00	0.00	−2.15	2.15
Facial expression	−0.08	−0.14	−1.25	1.08	0.50	0.14	−6.61	7.61
Eye contact	0.16	0.30	−0.90	1.21	0.60	0.18	−5.98	7.18

** p < .0001;

* p < .001;

ˆ p < .05

#### ASD Outcomes

Physical avoidance in the last hour but not the first minute significantly predicted ASD outcomes. We found that both a higher mean level (*B* = 5.84(1.67), *z* = 3.51, *p* < 0.001, CI[2.58, 9.12]), and a trajectory of increasing physical avoidance (*B* = 7.93(3.66), *z* = 2.17, *p* = 0.030, CI[0.76,15.12]) during the last hour of interaction predicted more severe ASD symptomology at outcome. We also found that both a higher mean level (*B* = 3.16(0.31), *z* = 10.12, *p* < 0.001, CI[2.55,3.77]) and a trajectory of increasing facial expressions of social wariness (*B* = 51.47(0.15), *z* = 355.02, *p* < 0.001 CI[51.19, 51.75]) during the first minute of interaction predicted more severe ASD symptomology at outcome. However, only the mean level, not the trajectory, of facial expression during the last hour of interaction (*B* = 8.51(2.50), *z* = 3.41, *p* = 0.001, CI[3.61,13.40]) predicted ASD symptomology. Finally, a higher mean level (*B* = 8.15(2.52), *z* = 3.23, *p* < 0.001, CI[3.20, 13.10]) of avoidant eye contact during the first minute of interaction predicted more severe ASD symptomology at outcome, whereas the trajectory did not. However, both a higher mean level (*B* = 10.05(2.02), *z* = 4.97, *p* < 0.001, CI[6.08, 14.02]) and a trajectory of increasing avoidance of eye contact (*B* = 8.22(2.21), *z* = 3.72, *p* < 0.001, CI[3.90, 12.55]) during the last hour of interaction predicted more severe ASD symptomology at outcome.

#### ADHD Outcomes

For ADHD, only the trajectory of increasing physical avoidance during the first minute of social interaction predicted decreased ADHD symptomology at outcome (*B* = −10.79(3.73), *z* = −2.89, *p* = 0.004, CI[−18.10, −3.47]). No other SAS variables predicted ADHD outcomes.

#### Anxiety Outcomes

For Anxiety, only the trajectory of increasing physical avoidance during the first minute of social interaction predicted decreased anxiety symptomology at outcome (*B* = −11.33(5.47), *z* = 2.07, *p* = 0.038, CI[−22.1, −0.61]), No other SAS domains predicted Anxiety Outcomes.

### Results Summary

Collectively, elevated physical avoidance, increased facial expressions of avoidance, and reduced eye contact were all predictors of ASD, ADHD, or anxiety outcomes. However, the relationships were dependent on the timing of the interaction (i.e., first minute or last hour) and parameter (i.e., average level or trajectory) with increased social avoidance predicting elevated ASD severity and, surprisingly, it also predicted reduced ADHD and anxiety severity at outcome. For ASD outcomes, we found that elevated physical avoidance, increased facial expressions of avoidance and reduced eye contact all signaled risk for the later onset of more severe ASD symptoms. Specifically, elevated levels of physical movement during the final hour and elevated levels of facial shyness and avoidant eye contact during both the first minute and final hour were associated with increased severity of ASD features. A trajectory of increased avoidance in physical movement and avoidant eye contact during the last hour and for the first minute for facial shyness also were associated with increased severity of ASD outcomes. And, while social avoidance during initial social encounters provided some signal for the later onset of ASD symptoms, it was elevated social avoidance during social encounters with familiar people that reflected the strongest indicator of more severe ASD symptomology. In contrast, only trajectories of increasing social avoidance during initial interactions were associated with reduced severity of ADHD and anxiety outcomes.

## Discussion

Psychiatric disorders are common and impairing in children, and efforts to increase understanding of the prodromal and predictive features of these disorders represent significant impact given the known benefits of early intervention. Given the high rates of ASD, ADHD and anxiety in males with FXS, studies of the developmental trajectories of symptoms associated with these psychiatric disorders can impact the timing and targets for intervention. This work has implications not only for individuals with FXS but also for those who exhibit features of ASD, ADHD and anxiety of known or unidentified etiology. Because of the high rates of comorbidity and the monogenic etiology of FXS, this population provides a saturated model for risk that includes more “signal” for detecting meaningful associations than can be observed in studies of the general population (47). The overall aim of this study was to document the relationship of social avoidance to ASD, ADHD and anxiety outcomes in males with FXS across a critical developmental period when symptoms and diagnoses are known to emerge. To accomplish this, we employed the SAS, an experimental observation scale that incorporates multiple dimensions of social avoidance within a temporal framework.

Based on our results, increased social avoidance across infancy and preschool predicted elevated severity of ASD symptoms but reduced ADHD and anxiety symptoms in males with FXS. A nuanced set of relationships was apparent with ASD predicted by specific social avoidance features that were entirely unique from those that predicted ADHD and anxiety outcomes. Surprisingly, identical predictors (trajectories of physical avoidance during initial interaction) emerged for both ADHD and anxiety outcomes. The trajectory of increasing social avoidance appears to be one of the most salient features as it predicted ASD, ADHD, and anxiety outcomes whereas an elevated level of social avoidance only predicted ASD features. However, it was the trajectory of initial interactions with unfamiliar people at the beginning of the assessment (first minute) that predicted reduced ADHD and anxiety whereas the trajectory of interactions with both unfamiliar people (beginning of the assessment) and people who had become more familiar by the end of the assessment (last hour) predicted ASD. These findings highlight the importance of capturing social avoidance across multiple domains and the value of including both initial responses to unfamiliar people as well as responses to people who have become familiar over the course of the assessment. Overall, these results suggest that elevated social avoidance emerging during infancy may be a salient marker for the onset for ASD and of reduced risk for ADHD and anxiety in males with FXS.

The finding that multiple indicators of social avoidance across the infant and preschool years' signal risk for the emergence of elevated ASD symptoms is of great importance. ASD is one of the most prevalent and impairing psychiatric disorders associated with FXS, and FXS is the leading identified genetic cause of ASD (22). Despite the importance of studying the association of ASD and FXS, few studies have identified early markers of ASD in FXS and most of these are cross-sectional. This focus of research is becoming increasingly studied, however, with recent reports indicating that social communication deficits (43, 48), motor atypicalities and delays (42, 49) along with gaze avoidance (50) and reduced escape during social challenges (51) may be detectable within the first years of life and serve as a signal for ASD risk in males with FXS. Here, we add that elevated levels and trajectories of increasing social avoidance also serve as risk markers for ASD in the largest longitudinal sample of infants and preschool-aged males with FXS to date.

Trajectories of social avoidance across infancy and preschool were also associated with ADHD and anxiety symptom outcomes, however, in the opposite direction to that expected with trajectories of elevated physical avoidance during initial social interactions predicting lower ADHD and anxiety symptom severity at outcome. These relationships were unexpected as we anticipated no relationship with ADHD as social impairment is an associated, but not core, feature of ADHD. The unexpected direction of the relationship could signal strong emotion regulation skills in these children. Per this interpretation, children who have less severe ADHD and anxiety symptom outcomes may have learned to manage their emotions by physically retreating from the source of stress, in this case, the approach of an unfamiliar person. This finding could also suggest a developmental effect in that children with less severe ADHD and anxiety outcomes may respond to social challenge differently across age with increasing physical avoidance of novel social partners more likely at preschool age with less physical avoidance during the infant and toddler years (52).

The results reported here are consistent with a recent study, which identified unique and distinct early-life predictors of ASD and ADHD, but are inconsistent with the same study documenting overlap in predictors of ASD and anxiety (53). Specifically, this recent study reported that shyness was associated with both anxiety and ASD. Here, differences in the relationship of social avoidance to the three psychiatric outcomes likely reflect the nature of the different disorders and the developmental period we have focused on. Increased severity of ASD features was associated with a number of aspects of social avoidance including all three dimensions (physical movement, facial expression, and eye contact) as well as during both initial and sustained social interactions (first minute and last hour). The pervasiveness of social avoidance associated with ASD likely reflects an aloof presentation which is often associated with non-syndromic ASD profiles (54). In contrast, reduced ADHD and anxiety symptoms align with an acute, and potentially adaptive, pattern of social avoidance only during initial social encounters. Children who do not have elevated ADHD or anxiety symptoms are more likely to have strong emotion regulation skills and for those skills to develop and increase across the preschool period (55, 56). Thus, the pattern of walking away from a potentially stressful social encounter as an adaptive response aligns with the nature of these disorders in that children with elevated ADHD might have little social avoidance and more impulsivity (57). And, those with elevated anxiety might be more likely to demonstrate initial social avoidance but to “warm up” over time.

One of the most important findings of this study is that ASD, ADHD and anxiety symptoms relate inconsistently to social avoidance behaviors, providing new insight toward the debate of independence or overlap among these disorders in FXS and other disorders (i.e., ASD). As noted above, ASD was the only disorder in which elevated social avoidance predicted increased severity of symptoms. In fact, increased social avoidance predicted reduced symptom severity for ADHD and anxiety. Thus, elevated social avoidance is a trait that uniquely predicts ASD and not ADHD or anxiety despite social avoidance being a feature of all three disorders. Our preliminary correlational analyses confirm this conclusion as ASD was not related to either ADHD or anxiety. This finding regarding the apparent independence across these disorders is particularly striking considering the high prevalence of 48.72 and 56.41% for ASD and ADHD who are above the clinical cut-offs, respectively. These rates are generally consistent with extant literature (15, 17). In contrast, only 7.70% of males with FXS were above the clinical cutoff for anxiety which may have constrained our power to detect a relationship between anxiety and trajectories of social avoidance. The rate of anxiety in our sample appears low compared to the fact that over 85% of older males with FXS meet diagnostic criteria for anxiety (18). However, no studies exist that document the early presentation of anxiety in FXS so the validity of its prevalence in our sample is unknown.

This study has a number of important clinical implications. First, we demonstrated that both the level and trajectory of social avoidance show meaningful individual differences in the expression of ASD, ADHD, and anxiety symptom outcomes in males with FXS. This suggests that the nature and function of social avoidance is complex, and caution should be taken to attend to the nuanced profile of social avoidance to ensure diagnostic accuracy with developmental and temporal aspects of social avoidance carefully considered. Specifically, both initial and prolonged social avoidance should be integrated into differential diagnoses, and this practice rests on clinical knowledge and observation as some tools (e.g., ADOS-2, CBCL) may not be sufficiently sensitive to these clinical factors. Second, our findings confirm that social avoidance manifests very early in development, and we extend initial work in this area by demonstrating that early trajectories of social avoidance may index risk for ASD while signaling resilience or a protective factor for ADHD and anxiety in young males with FXS. This information is critical to inform the timing and targets for treatment.

Despite inclusion of the largest sample of males with FXS to date in a study focused on social avoidance and integration of multiple outcomes with repeated measures, there are a number of limitations. These include failure to include females and reliance on behavioral ratings without biomarkers. We also targeted the first years of life as a critical developmental period as our focus was on early signs and symptoms vs. diagnoses. However, the focus on this early childhood period likely constrained our ability to fully detect the relationship of social avoidance to anxiety given that symptoms of anxiety emerge later in development than ASD and ADHD symptoms. Also, with regard to our models, the average amount of assessments available across children was three *(SD* = 1.04), with some children having as many as five assessments, and some children having as few as one assessment. Because of this range, we modeled our trajectories using simple linear trends; however, in reality, these trends are not likely entirely linear. Thus, our understanding of how developmental changes in social avoidance influences later outcomes is constrained to linear trends (i.e., the expected outcome if children's social avoidance improves or declines, on average, over time), rather than more nuanced profiles that could be estimated with additional data. These are important factors for future work, as is inclusion of a contrast group to see if these findings generalize to other clinical or non-clinical groups (e.g., non-syndromic ASD or low-risk controls).

## Ethics Statement

This study was carried out in accordance with the recommendations of Human Subjects Research University of South Carolina Institutional Review Board and the Institutional Review Board at University of North Carolina at Chapel Hill, with written informed consent from all subjects. All subjects gave written informed consent in accordance with the Declaration of Helsinki. The protocol was approved by the University of South Carolina Institutional Review Board and the Institutional Review Board at the University of North Carolina at Chapel Hill.

## Author Contributions

JR conceptualized the study and oversaw data collection and provided leadership for producing the paper. EW and SM analyzed the data with consultation from BT and AH. HC played a key role in writing the paper with input from AH. SO, DR, and AB wrote sections of the paper.

### Conflict of Interest Statement

The authors declare that the research was conducted in the absence of any commercial or financial relationships that could be construed as a potential conflict of interest. The handling editor declared a shared affiliation, though no other collaboration, with several of the authors, JR, EW, AH, SM, SO, AB.
